# IPAD-DB: a manually curated database for experimentally verified inhibitors of proteins associated with Alzheimer’s disease

**DOI:** 10.1093/database/baae048

**Published:** 2024-06-12

**Authors:** Chong Peng, Xiaofeng Liu, Xiangbo Meng, Congge Chen, Xinming Wu, Lin Bai, Fuping Lu, Fufeng Liu

**Affiliations:** Key Laboratory of Industrial Fermentation Microbiology, Ministry of Education, Tianjin 300457, P. R. China; Tianjin Key Laboratory of Industrial Microbiology, Tianjin 300457, P. R. China; College of Biotechnology, Tianjin University of Science and Technology, Tianjin 300457, P. R. China; College of Biotechnology, Tianjin University of Science and Technology, Tianjin 300457, P. R. China; College of Biotechnology, Tianjin University of Science and Technology, Tianjin 300457, P. R. China; College of Biotechnology, Tianjin University of Science and Technology, Tianjin 300457, P. R. China; College of Biotechnology, Tianjin University of Science and Technology, Tianjin 300457, P. R. China; College of Biotechnology, Tianjin University of Science and Technology, Tianjin 300457, P. R. China; Key Laboratory of Industrial Fermentation Microbiology, Ministry of Education, Tianjin 300457, P. R. China; Tianjin Key Laboratory of Industrial Microbiology, Tianjin 300457, P. R. China; College of Biotechnology, Tianjin University of Science and Technology, Tianjin 300457, P. R. China; Key Laboratory of Industrial Fermentation Microbiology, Ministry of Education, Tianjin 300457, P. R. China; Tianjin Key Laboratory of Industrial Microbiology, Tianjin 300457, P. R. China; College of Biotechnology, Tianjin University of Science and Technology, Tianjin 300457, P. R. China

## Abstract

Alzheimer’s disease (AD) is a universal neurodegenerative disease with the feature of progressive dementia. Currently, there are only seven Food and Drug Administration-approved drugs for the treatment of AD, which merely offer temporary relief from symptom deterioration without reversing the underlying disease process. The identification of inhibitors capable of interacting with proteins associated with AD plays a pivotal role in the development of effective therapeutic interventions. However, a vast number of such inhibitors are dispersed throughout numerous published articles, rendering it inconvenient for researchers to explore potential drug candidates for AD. In light of this, we have manually compiled inhibitors targeting proteins associated with AD and constructed a comprehensive database known as IPAD-DB (Inhibitors of Proteins associated with Alzheimer’s Disease Database). The curated inhibitors within this database encompass a diverse range of compounds, including natural compounds, synthetic compounds, drugs, natural extracts and nano-inhibitors. To date, the database has compiled >4800 entries, each representing a correspondent relationship between an inhibitor and its target protein. IPAD-DB offers a user-friendly interface that facilitates browsing, searching and downloading of its records. We firmly believe that IPAD-DB represents a valuable resource for screening potential AD drug candidates and investigating the underlying mechanisms of this debilitating disease. Access to IPAD-DB is freely available at http://www.lamee.cn/ipad-db/ and is compatible with all major web browsers.

**Database URL**: http://www.lamee.cn/ipad-db/

Key points

- We have manually compiled inhibitors targeting proteins associated with Alzheimer’s disease and constructed a comprehensive database known as IPAD-DB (Inhibitors of Proteins associated with Alzheimer’s Disease Database).
- The IPAD-DB comprises a comprehensive collection of 4804 manually curated associations between inhibitors and target proteins sourced from various publications. The inhibitors are categorized into five distinct classes: natural compounds, synthetic compounds, drugs, natural extracts and nano-inhibitors.
- We firmly believe that IPAD-DB represents a valuable resource for screening potential AD drug candidates and investigating the underlying mechanisms of this debilitating disease.

## Introduction

Alzheimer’s disease (AD) is a systemic neurodegenerative disease characterized by progressive dementia (1). It initiates with mild cognitive impairment and, as the disease progresses with age, leads to the degeneration of specific nerve cells involved in memory, language and cognition. This results in an inability to communicate or respond to the environment, putting patients at risk of severe harm and death (2, 3). It is estimated that ∼416 million individuals worldwide are afflicted by varying stages of AD, encompassing AD dementia, prodromal AD and preclinical AD, accounting for ∼22% of the global population aged ≥50 years (4).

The pathological manifestations of AD are intricate, including the formation of senile plaques composed of amyloid-β (Aβ) protein, intracellular neurofibrillary tangles formed by abnormal phosphorylation of tau protein, synaptic dysfunction and neurotrophy (5). Despite extensive research efforts, the underlying mechanisms of driving these pathological changes remain elusive, and the reasons for their occurrence are not fully understood. Nonetheless, it is widely accepted that several factors play crucial roles in the pathophysiology of the disease, including acetylcholine deficiency, Aβ protein deposition, tau protein phosphorylation, oxidative stress and metal homeostasis imbalance (6). For decades, researchers have endeavored to develop therapeutic agents for AD based on their comprehension of the targets implicated in AD-associated pathological alterations. Presently, the two primary types of AD medications encompass acetylcholinesterase inhibitors (AChEIs) and *N*-methyl-d-aspartate (NMDA) receptor antagonists. AChEI helps with information transmission between nerve cells, while NMDA receptor antagonists aim to mitigate neuronal damage caused by elevated glutamate levels. However, these medications merely offer symptomatic relief and are often accompanied by significant adverse effects. Thus, there persists an imperative need for the advancement of AD drug development research.

The identification of compounds capable of inhibiting pathogenic proteins associated with AD and the subsequent development of corresponding therapeutic drugs have emerged as significant areas of research. However, the inhibitors that have been identified are scattered throughout various literature sources, resulting in fragmented information that hinders systematic analysis and calculation of AD-related protein inhibitors. To address this issue, we have created a meticulously curated database, known as the Inhibitors of Proteins Associated with Alzheimer’s Disease Database (IPAD-DB), which exclusively contains experimentally verified inhibitors. While existing databases such as AlzData (7, 8), Alzheimer’s Disease Neuroimaging Initiative (9), Alzheimer’s Disease Genetics Consortium (available at https://www.adgenetics.org/), Alzforum (10) and National Alzheimer’s Coordinating Center (11) primarily focus on AD-related clinical, imaging and genetic data, including genes, mutations, proteins and biomarkers, IPAD-DB places greater emphasis on inhibitors targeting AD-related proteins such as Aβ protein, AChE, Tau and BACE-1. To the best of our knowledge, IPAD-DB is currently the only database offering such comprehensive data. Pharmacologists can employ IPAD-DB to screen potential drug candidates for AD, while researchers, particularly those utilizing artificial intelligence technology for the rational design of inhibitors, can delve into the pathogenesis of AD by analyzing compounds that effectively inhibit AD-related proteins.

## Materials and methods

### Data collection and organization

The initial step in establishing IPAD-DB is the meticulous compilation of a curated list of experimentally verified inhibitors of proteins associated with AD from published literature. Our literature search was conducted using a spectrum of keywords including ‘Alzheimer’s disease’, ‘treatment’, ‘inhibition’, ‘aggregation’ and ‘Amyloid beta protein or tau protein or BACE or AChE’ in the PubMed (12) and Web of Science databases (up to July 2023). It is necessary to note that there is a considerable overlap in the articles indexed on the PubMed and Web of Science platforms. We initially conducted our search on the Web of Science database, saving the articles for later review. We then repeated the same search on PubMed, comparing the entries from both databases. If an article was found in the earlier collection, we excluded it to create a non-redundant collection of literature. Moreover, during the data collection phase, we reviewed the abstracts to assess the articles’ relevance to AD inhibitors, collecting data on those that were pertinent. The gathered inhibitors were then categorized into five groups: natural compounds, synthetic compounds, drugs, natural extracts and nano-inhibitors. A total of 2078 inhibitors have been amassed from 1128 literature sources. Indeed, inhibitors are inherently intertwined with their protein targets, necessitating a paired existence. Therefore, the combination of inhibitor and target protein is included as a basic entry in the database. Because some literature studies discussed the inhibition of single inhibitor on multiple proteins, the final number of entries in the database is 4804.

**Figure 1.**
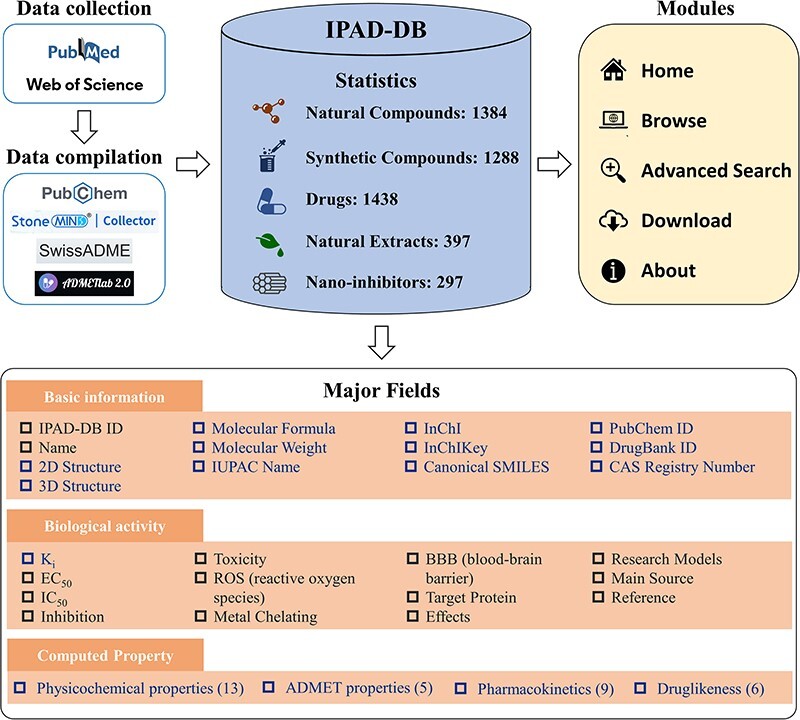
The architecture and content of IPAD-DB. In the major fields’ module, the black font is the information listed for all five inhibitors. The blue font is information unique to natural compounds, synthetic compounds and drugs.

For each of the five groups of inhibitors, we extracted their names, biological activity data (including Ki, EC50 and IC50), toxicity, reactive oxygen species (ROS), metal chelation, blood–brain barrier permeability (BBB), target protein, effect, research models, main source and literature URL from the collected literature. In the case of natural compounds, synthetic compounds and drugs, their names were cross-referenced with the PubChem database (13) to gather fundamental information about the compound, such as molecular formula, molecular weight, structure (2D and 3D), IUPAC name, InChI, InChIKey and canonical SMILES. The collected inhibitors are also linked to existing databases such as DrugBank (14) and CAS Common Chemistry (15) for further investigation. In instances where molecules could not be retrieved from PubChem, we utilized StoneMIND Collector software (StoneMIND Release 2021-9, StoneMIND Collector, Beijing StoneWise Technology Co. Ltd, CN, 2021) to map the compound’s 2D structure based on the provided information in the literature. These structures were then converted to IUPAC name, InChI, InChIKey and Canonical SMILES formats and stored in IPAD-DB. Based on the Canonical SMILES of inhibitors, we used two online platforms, namely SwissADME (16) and ADMETlab 2.0 (17), to compute the physicochemical characteristics; absorption, distribution, metabolism, excretion and toxicity (ADMET) properties; pharmacokinetic predictions and druglikeness of the inhibitors. The detailed architecture of IPAD-DB is illustrated in Figure 1.

### Database implementation

To ensure efficient data retrieval and facilitate maintenance, the IPAD-DB employed SQLite as the database engine. The website itself was constructed using the Django framework, with webpage design executed through HTML and CSS. In addition to keyword-based searches, the ‘Advanced Search’ page of the database also offers a ‘Structure Search’ module. We have integrated the JSME editor (18) to allow users to sketch a query molecule. The submitted molecule is then converted to SMILES format and assessed for molecular similarity to the inhibitors stored in IPAD-DB. RDKit (19) was employed to calculate molecular similarity.

## Results

### Database description

The IPAD-DB comprises a comprehensive collection of 2078 manually curated inhibitors sourced from various publications. Considering that the literature may analyze the correlation between inhibitors and more than one AD-related protein, we have treated each combination of an inhibitor and its target protein as a fundamental entry within the database. The database now contains 4804 entries, covering 134 target proteins in total. Entries pertaining to Aβ are the most numerous, amounting to 1978. Following closely is acetylcholinesterase (AChE), with 749 corresponding entries. There are also proteins with fewer related studies, such as Aβ17-37, Aβ1-28 and Aβ42 oligomerization, which have only one inhibitor associated with them. On the main page of the database, we have tabulated the number of inhibitors corresponding to target proteins with more than two inhibitors. We have organized the entries according to the type of inhibitors, dividing them into five classes: natural compounds, synthetic compounds, drugs, natural extracts and nano-inhibitors. Moreover, we have assigned them IDs starting with the initial letters C, S, D, E and N, respectively. For each type of inhibitor, we offer extensive annotations obtained through meticulous compilation and integration, utilizing a wide array of publicly available resources. These annotations encompass six key aspects, including (i) basic information, (ii) biological activity data, (iii) physicochemical properties, (iv) ADMET properties, (v) pharmacokinetic prediction and (vi) drug similarity prediction. The first part is a brief introduction of the inhibitor, which includes the assigned IPAD-DB ID and inhibitor name retrieved from the literature. In the case of natural compounds, synthetic compounds and drugs, the basic information of inhibitors also includes molecular formula, molecular weight, structure (2D and 3D), IUPAC name, InChI, InChIKey, canonical SMILES and the registration number that can be linked to the PubChem, DrugBank and CAS Common Chemistry. The biological activity data comprise K_i_, EC_50_, IC_50_, toxicity, ROS, metal chelation, BBB, target protein, effect, research models and main source. These data were obtained from the literature. Based on the canonical SMILES of the inhibitors, 13 important physicochemical properties were calculated using SwissADME and ADMETlab 2.0 online prediction websites. These properties encompass molecular weight, number of heavy atoms (Hac), volume, density, number of rings (nRing), number of atoms in the largest ring (MaxRing), number of heteroatoms (nHet), formal charge (fChar), number of rigid bonds (nRig), flexibility, stereo centers, Log of the aqueous solubility (logS) and Log of the partition coefficient at PH7.4 (LogD).

In practice, the inadequate ADMET of drugs are significant factors contributing to the failure of drug development. The assessment of ADMET characteristics is pivotal in the drug discovery process, as these indicators can be utilized to screen out unfavorable compounds in the early stages of drug development. We have calculated five ADMET properties, including water partition coefficient (logP), topological polar surface area, hydrogen bond acceptors/donors and number of rotatable bonds. Finally, to evaluate the pharmacokinetics and druglikeness of the inhibitors, we used SwissADME to calculate multiple attributes, including GI absorption, BBB permeant, P-gap substrate, CYP1A2 inhibitor, CYP2C19 inhibitor, CYP2C9 inhibitor, CYP2D6 inhibitor, CYP3A4 inhibitor and skin permeation in pharmacokinetics. Lipinski filter, Ghose filter, Veber filter, Egan filter, Muegge filter and bioavailability score were evaluated. All datasets and annotations are freely accessible to all users.

### Database usage

This database offers a user-friendly interface, featuring a global navigation bar positioned at the top of each page, facilitating seamless switching between different pages for users. By simply clicking on the designated text box on the main page, users can readily access concise information pertaining to each category of inhibitor. Moreover, hovering the cursor over the ‘Browse’ button triggers a drop-down menu, offering users another way to effortlessly explore the brief information page associated with each inhibitor category. The concise information page for natural products, synthetic compounds and drugs presents the number, name, target protein, molecular formula, 2D structure and reference link for each inhibitor. The page for natural extracts and nano-inhibitors provides the number, name, target protein, research model and reference link. On the individual details page for natural compounds, synthetic compounds and drugs, all six aspects of annotations are meticulously listed. The collected inhibitors are cross-referenced with existing databases to facilitate further investigation. However, due to the fact that natural extracts and nano-inhibitors are mixtures lacking a singular structure, certain physical and chemical properties cannot be predicted or provided.

IPAD-DB offers users the convenience of both text- and structure-based search methods. To conduct a text-based search, users may input the name of the inhibitor, the targeted protein, the molecular SMILES or the IPAD-DB ID into the designated search box. Subsequently, they can select the type of inhibitor and the category of keywords. On the other hand, for a structure-based search, users have the option to utilize the JSME (18) editor to sketch a query molecule, extract its SMILES representation and explore the database for compounds with analogous structures. The comprehensive collection of inhibitor annotations and structures can be conveniently obtained through the ‘Download’ page for batch downloading. The IPAD-DB database is readily accessible online at http://www.lamee.cn/ipad-db/ and does not require any registration.

## Conclusion and discussions

The development of effective therapeutic interventions for AD remains a significant challenge. The limited number of Food and Drug Administration-approved drugs for AD only provides temporary relief from symptoms, highlighting the need for the identification of novel inhibitors that can target proteins associated with the disease. The present study has successfully established a comprehensive database named IPAD-DB, housing a diverse collection of inhibitors targeting proteins associated with AD. The database includes various types of compounds, ranging from natural compounds to synthetic compounds, drugs, natural extracts and nano-inhibitors. With a user-friendly interface, IPAD-DB facilitates effortless browsing, searching and downloading of records within the database. This resource will greatly facilitate the screening of potential AD drug candidates and the investigation of the underlying mechanisms of the disease. By providing a centralized and accessible platform for researchers, IPAD-DB has the potential to accelerate the discovery and development of effective treatments for AD.

Furthermore, the availability of IPAD-DB to the scientific community free of charge ensures that researchers from all backgrounds and institutions can access and utilize this valuable resource. This inclusivity promotes collaboration and knowledge sharing, ultimately fostering advancements in AD research.

However, it is important to acknowledge the limitations of IPAD-DB at its current stage. First, although the database contains a substantial number of inhibitors, there may still be some potential inhibitors that have not been included. Second, while we have meticulously curated references from high-impact, peer-reviewed journals to ensure a robust foundation for our database, practical limitations have precluded us from replicating experiments for each inhibitor individually, consequently not permitting a comprehensive verification of the empirical evidence’s reliability. Given the variability in experimental methodologies across distinct publications and the potential incompleteness of inhibitor details provided, researchers should exercise caution when evaluating and validating the activity and reliability of inhibitors within IPAD-DB.

Moving forward, IPAD-DB can serve as a foundation for future studies in the field of AD drug discovery. Researchers can utilize the database to identify novel inhibitors, explore their mechanisms of action and potentially develop new therapeutic strategies. Additionally, the continuous expansion and updating of IPAD-DB with new inhibitors and relevant information will enhance its utility and value to the scientific community.

In conclusion, IPAD-DB provides a comprehensive and accessible resource for the identification and investigation of inhibitors targeting proteins associated with AD. The potential applications of IPAD-DB in accelerating the development of effective AD treatments and advancing our understanding of the disease are promising. Researchers are encouraged to utilize this database and contribute to its growth, ultimately leading to improved outcomes for patients suffering from AD.

## Data Availability

IPAD-DB is freely available online at http://www.lamee.cn/ipad-db/.

## References

[1] Blennow K. , de Leon M.J. and Zetterberg H. (2006) Alzheimer’s disease. *Lancet*, 368, 387–403. 16876668 10.1016/S0140-6736(06)69113-7

[2] Gaugler J. , James B., Johnson T. et al. (2022) 2022 Alzheimer’s disease facts and figures. *Alzheimers Dementia*, 18, 700–789. 10.1002/alz.12638 35289055

[3] Nichols E. , Steinmetz J.D., Vollset S.E. et al. (2022) Estimation of the global prevalence of dementia in 2019 and forecasted prevalence in 2050: an analysis for the Global Burden of Disease Study 2019. *Lancet Public Health*, 7, E105–E125. 34998485 10.1016/S2468-2667(21)00249-8 PMC8810394

[4] Gustavsson A. , Norton N., Fast T. et al. (2023) Global estimates on the number of persons across the Alzheimer’s disease continuum. *Alzheimers Dementia*, 19, 658–670. 10.1002/alz.12694 35652476

[5] Krance S.H. , Cogo-Moreira H., Rabin J.S. et al. (2019) Reciprocal predictive relationships between amyloid and tau biomarkers in Alzheimer’s disease progression: an empirical model. *J. Neurosci*., 39, 7428–7437. 31350262 10.1523/JNEUROSCI.1056-19.2019 PMC6759020

[6] Savelieff M.G. , Nam G., Kang J. et al. (2019) Development of multifunctional molecules as potential therapeutic candidates for Alzheimer’s disease, Parkinson’s disease, and amyotrophic lateral sclerosis in the last decade. *Chem. Rev*., 119, 1221–1322. 30095897 10.1021/acs.chemrev.8b00138

[7] Xu M. , Zhang D.-F., Luo R. et al. (2018) A systematic integrated analysis of brain expression profiles reveals YAP1 and other prioritized hub genes as important upstream regulators in Alzheimer’s disease. *Alzheimers Dementia*, 14, 215–229. 10.1016/j.jalz.2017.08.012 28923553

[8] Zhang D.F. , Fan Y., Xu M. et al. (2019) Complement C7 is a novel risk gene for Alzheimer’s disease in Han Chinese. *Natl. Sci. Rev*., 6, 257–274. 31032141 10.1093/nsr/nwy127 PMC6477931

[9] Petersen R.C. , Aisen P.S., Beckett L.A. et al. (2010) Alzheimer’s disease neuroimaging initiative (ADNI) clinical characterization. *Neurology*, 74, 201–209. 20042704 10.1212/WNL.0b013e3181cb3e25 PMC2809036

[10] Clark T. and Kinoshita J. (2007) Alzforum and SWAN: the present and future of scientific web communities. *Briefings Bioinf*., 8, 163–171. 10.1093/bib/bbm012 17510163

[11] Beekly D.L. , Ramos E.M., van Belle G., et al. (2004) The National Alzheimer’s Coordinating Center (NACC) Database—an Alzheimer disease database. *Alzheimer Dis Assoc. Disord*., 18, 270–277. 15592144

[12] Sayers E.W. , Bolton E.E., Brister J. et al. (2022) Database resources of the National Center for Biotechnology Information in 2023. *Nucleic Acids Res*., 51, D29–D38. 10.1093/nar/gkac1032 PMC9825438 36370100

[13] Kim S. , Chen J., Cheng T. et al. (2023) PubChem 2023 update. *Nucleic Acids Res*., 51, D1373–D1380. 36305812 10.1093/nar/gkac956 PMC9825602

[14] Wishart D.S. , Feunang Y.D., Guo A.C. et al. (2018) DrugBank 5.0: a major update to the DrugBank database for 2018. *Nucleic Acids Res*., 46, D1074–D1082. 29126136 10.1093/nar/gkx1037 PMC5753335

[15] Jacobs A. , Williams D., Hickey K. et al. (2022) CAS Common Chemistry in 2021: expanding access to trusted chemical information for the scientific community. *J. Chem. Inf. Model*., 62, 2737–2743. 35559614 10.1021/acs.jcim.2c00268 PMC9199008

[16] Daina A. , Michielin O. and Zoete V. (2017) SwissADME: a free web tool to evaluate pharmacokinetics, drug-likeness and medicinal chemistry friendliness of small molecules. *Sci. Rep*., 7, 42717. 10.1038/srep42717 PMC5335600 28256516

[17] Xiong G.L. , Wu Z., Yi J. et al. (2021) ADMETlab 2.0: an integrated online platform for accurate and comprehensive predictions of ADMET properties. *Nucleic Acids Res*., 49, W5–W14. 33893803 10.1093/nar/gkab255 PMC8262709

[18] Bienfait B. and Ertl P. (2013) JSME: a free molecule editor in JavaScript. *J. Cheminf*., 5, 24. 10.1186/1758-2946-5-24 PMC3662632 23694746

[19] Lovric M. , Molero J.M. and Kern R. (2019) PySpark and RDKit: moving towards big data in cheminformatics. *Mol. Inform*., 38, e1800082. 10.1002/minf.201800082 30844132

